# Auditory Brainstem Responses to Continuous Natural Speech in Human Listeners

**DOI:** 10.1523/ENEURO.0441-17.2018

**Published:** 2018-02-09

**Authors:** Ross K. Maddox, Adrian K. C. Lee

**Affiliations:** 1 Department of Biomedical Engineering, University of Rochester, Rochester, NY 14627; 2 Department of Neuroscience, University of Rochester, Rochester, NY 14642; 3 Del Monte Institute for Neuroscience, University of Rochester, Rochester, NY 14642; 4 Institute for Learning and Brain Sciences, University of Washington, Seattle, WA 98195; 5 Department of Speech and Hearing Sciences, University of Washington, Seattle, WA 98105

**Keywords:** auditory brainstem response, deconvolution, electroencephalography, encoding model, speech

## Abstract

Speech is an ecologically essential signal, whose processing crucially involves the subcortical nuclei of the auditory brainstem, but there are few experimental options for studying these early responses in human listeners under natural conditions. While encoding of continuous natural speech has been successfully probed in the cortex with neurophysiological tools such as electroencephalography (EEG) and magnetoencephalography, the rapidity of subcortical response components combined with unfavorable signal-to-noise ratios signal-to-noise ratio has prevented application of those methods to the brainstem. Instead, experiments have used thousands of repetitions of simple stimuli such as clicks, tone-bursts, or brief spoken syllables, with deviations from those paradigms leading to ambiguity in the neural origins of measured responses. In this study we developed and tested a new way to measure the auditory brainstem response (ABR) to ongoing, naturally uttered speech, using EEG to record from human listeners. We found a high degree of morphological similarity between the speech-derived ABRs and the standard click-evoked ABR, in particular, a preserved Wave V, the most prominent voltage peak in the standard click-evoked ABR. Because this method yields distinct peaks that recapitulate the canonical ABR, at latencies too short to originate from the cortex, the responses measured can be unambiguously determined to be subcortical in origin. The use of naturally uttered speech to measure the ABR allows the design of engaging behavioral tasks, facilitating new investigations of the potential effects of cognitive processes like language and attention on brainstem processing.

## Visual Abstract

**Figure:**
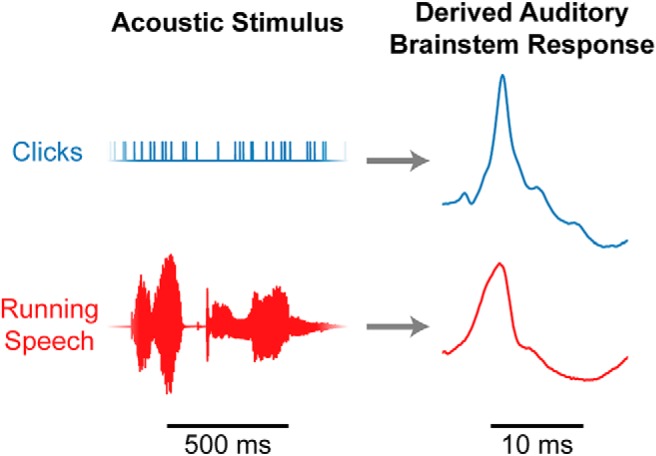

## Significance Statement

The brainstem is crucial to speech processing, yet a majority of speech studies have focused on the cortex. This is in large part because practical limitations have made elusive a paradigm for studying brainstem processing of continuous natural speech in human listeners. Here, we adapt methods that have been employed for studying cortical activity to the auditory brainstem. We measure the response to continuous natural speech and show that it recapitulates important aspects of the click-evoked response. The method also allows simultaneous investigation of cortical activity with no added recording time. This discovery paves the way for studies of speech processing in the human brainstem, including its interactions with higher order cognitive processes originating in the cortex.

## Introduction

When speech enters the ear and is encoded by the cochlea, it goes on to be processed by an ascending pathway that spans the auditory nerve, brainstem, and thalamus before reaching the cortex. Far from being relays, these subcortical nuclei perform a dazzling array of important functions, from sound localization (Grothe and Pecka, 2014) to vowel coding (Carney et al., 2015), making their function essential to understand. In humans, the primary method for measuring activity in subcortical nuclei is the auditory brainstem response (ABR): a highly stereotyped scalp potential in the first ∼10 ms following a very brief stimulus such as a click, recorded through electroencephalography (EEG; Burkard et al., 2006). The potential comprises components referred to as waves, given Roman numerals I–VII according to their latency. Individual waves have been tied to activity in specific parts of the ascending pathway: Wave I (∼2-ms latency) is driven by auditory nerve activity, Wave III (∼4 ms) by the cochlear nucleus, and Wave V (∼6 ms) principally by the lateral lemniscus (Møller et al., 1995). However, because the waves are so rapid, and the signal-to-noise ratio (SNR) so low, the ABR must be measured by presenting thousands of repeated punctate stimuli. Thus, while there are important neuroscience questions regarding how subcortical nuclei process natural stimuli like speech, or how they might be affected by cognitive processes through efferent feedback (Terreros and Delano, 2015), the practical limitations of the ABR paradigm make it primarily a clinical tool.

One common method for measuring the brainstem response to speech is the complex ABR (cABR; Skoe and Kraus, 2010). The cABR represents the averaged response to repetitions of a short-spoken syllable (e.g., a ∼40-ms “da”). The onset response can be analyzed in the time domain, but because the stimulus is longer than the response, ambiguity about the origin of response components arises for all but the earliest latencies. The voiced part of the speech elicits a frequency-following response (FFR) that can be analyzed in the frequency domain. The FFR has been shown to be primarily driven by the inferior colliculus of the brainstem, but it results from a mixture of sources including the superior olive (Smith et al., 1975) and also may include small contributions from the cortex (Coffey et al., 2016).

A different method, used for studying cortical activity, treats the auditory evoked potential as the impulse response of a linear system, which can be mathematically derived from known input and output signals (Aiken and Picton, 2008; Lalor et al., 2009; Lalor and Foxe, 2010; Ding and Simon, 2012a, 2012b). Continuous natural speech is presented (input) while EEG is recorded (output), and the brain’s response is calculated through linear regression. Rather than raw audio, the regressor (i.e., input) used is the amplitude envelope, which by construction contains no fast fluctuations, making it too slow for studying subcortical nuclei. A recent study aimed at the brainstem uses the cross-correlation of the speech stimulus’s fundamental waveform with the EEG recording (Forte et al., 2017). The response is a single peak with a latency of 9 ms but a relatively broad width. As with the FFR, interpreting this response is complicated by the likelihood that it is dominated by the inferior colliculus but still represents a mixture of sources.

Here, we measured auditory brainstem activity in response to natural speech using a new paradigm. The methods were based on cortical studies, with an important difference: the regressor was the rectified speech audio, meaning that fine structure was largely preserved. The speech-derived responses recapitulated important aspects of the click-evoked ABR, most notably in the presence of a distinct Wave V. The speech-derived Wave V latency and amplitude were both highly correlated with the click-evoked response across subjects, demonstrating common neural generators. Thus, by preserving the latencies of individual response components, the speech-derived ABR allows the experimenter to assess neural activity at separate stages along the auditory pathway.

The goal of this study was to develop a method for measuring the ABR to natural speech in experiments where clicks and other standard stimuli are disadvantageous, inappropriate, or impossible to use. The results show that it is possible to use natural speech stimuli to study speech processing in the human brainstem, paving the way for subcortical studies of attention, language, and other cognitive processes.

## Materials and Methods

### Experimental design and statistical analysis

Our goal was to measure the speech-derived ABR in human listeners and validate it against the click-evoked response. We first recorded click-evoked responses to pseudorandomly timed click trains and then validated them against the responses evoked by standard, periodic click trains. We then compared the speech-derived response to the pseudorandom click-evoked response. We validated by the speech-derived response by comparing its overall morphology and Wave V latency and amplitude to those of the click-evoked response.

All subjects’ click- and speech-derived responses were plotted individually. To compare the similarity of two responses from a single subject (e.g., the click-evoked response to the speech-derived response), Pearson’s product-moment correlation was used. The median and interquartile range of each distribution of correlation coefficients across subjects were reported, in addition to plotting the correlation histogram. Two distributions of correlation coefficients were compared using Wilcoxon’s signed-rank test for non-normal distributions.

### Subjects

All experiments were done under a protocol approved by the University of Washington Institutional Review Board. All subjects gave informed consent before participation, and were compensated for their time. We collected data from 24 subjects (17 females). The mean age was 27.8 years, with a SD of 6.9 and a range of 19–45. Subjects had normal hearing, defined as audiometric thresholds of 20 dB HL or better in both ears at octave frequencies ranging from 250–8000 Hz. All subjects identified English as their first language except for two, who identified a different language but had been speaking English daily for over 20 years.

### EEG recording

Scalp potentials were recorded with passive Ag/AgCl electrodes, with the positive and negative electrodes connected to a differential preamplifier (Brainvision LLC). The positive electrode was at location FCz in the standard 10–20 coordinate system. The negative (reference) electrode was clipped onto the subject’s left earlobe. The ground electrode was placed at Fpz. Data were high-pass filtered at 0.1 Hz during recording (additional filtering occurred offline).

Subjects were seated in a comfortable chair in a sound-treated room (IAC). They were not asked to attend the stimuli. Instead, they faced a computer monitor showing silent episodes of *Shaun the Sheep* (Starzak and Sadler, 2007), an animated show that has no talking, making subtitles unnecessary. They were first presented with 40 epochs of speech stimuli for calculating the speech ABR, and then were presented with 10 min of click stimuli (20 repetitions of a frozen 30-s epoch). All stimuli were presented over insert earphones (ER-2, Etymotic Research), which were plugged into a stimulus presentation system consisting of a real-time processor and a headphone amplifier (RP2.1 and HB7, respectively, Tucker Davis Technologies). Stimulus presentation was controlled with a python script using publicly available software (available at https://github.com/LABSN/expyfun).

### Speech stimuli

**Figure 1.**
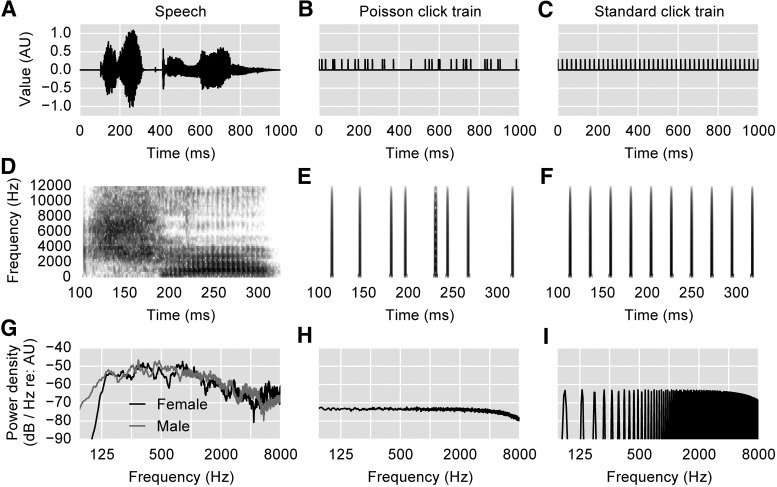
Acoustic stimuli. ***A–C***. Pressure waveforms for one second of speech, Poisson click train, and standard periodic click train, respectively. Vertical scale is plotted in arbitrary units (AU), but is consistent across plots. ***D–F***, Spectrograms of a smaller excerpt of the above stimuli, with darker colors corresponding to higher power. ***G–I***, PSD plots of the above stimuli, calculated from 30 s of data using Welch’s method with a segment length of 5.67 ms, segment overlap of 50%, and Hann window. Note that although the speech recordings were gently high-pass filtered at 1000 Hz, there remains plenty of power in the 125- to 1000-Hz range (***G***) and pitch information is clearly preserved (***D***; vertical striations between 200 and 300 ms correspond to individual glottal pulses).

Speech stimuli were taken from two audiobooks. The first was *A Wrinkle in Time* (L’Engle, 2012), read by a female narrator. The second was *The Alchemyst* (Scott, 2007), read by a male narrator. The audiobooks were purchased on compact disk and ripped to uncompressed wav files to avoid data compression artifacts. They were resampled to 24,414 Hz, the native rate of the RP2 presentation system. They were then processed so that any silent pauses in the speech longer than 0.5 s were truncated to 0.5 s. Because the ABR is principally driven by higher stimulus frequencies (Abdala and Folsom, 1995), the speech was gently high-passed with a first-order Butterworth filter with a cutoff of 1000 Hz and a slope of 6 dB/octave. The speech was still natural sounding and completely intelligible. This filter also helped to compensate for low-frequency spectral differences between the male and female narrator around their fundamental frequencies. After that, the speech was normalized to an average root-mean-square amplitude that matched that of a 1-kHz tone at 75 dB SPL. Figure 1*A*,*D*,*G* show the pressure waveform of the word “Thursday” spoken by the male narrator, the spectrogram of that word’s first syllable, and the power spectral density (PSD) of a 30-s segment of the female and male speech stimuli. It is evident from Figure 1*D* that the filtering did not affect the presence of pitch information (glottal pulses at the fundamental frequency are easily visible as vertical striations, even well below 1000 Hz), and from Figure 1*G* that the lowest speech formants were still present (plenty of energy remaining in 300- to 500-Hz region).

The audiobooks were then sectioned into epochs of 64 s, including a 1-s raised cosine fade-in and fade-out. The last four seconds of each epoch were repeated as the first four seconds of the next one, so that subjects could pick up where they left off in the story (if they were listening), meaning that 60 s of novel speech were presented in each epoch. The stimuli were not new to the subjects; before this passive listening task, they had completed a session using the same stimuli where they had to answer questions about the content they had just heard. Data from that task were for a different scientific question and do not appear here. These minute-long excerpts were presented in sequence, two from one story and then in alternating sets of four, finishing with two epochs from the second story. Speech stimuli were presented diotically.

### Click stimuli

Click stimuli were aperiodic trains of rarefaction clicks lasting 82 µs (representing two samples at the 24,414-Hz sampling rate, which was as close as possible to the standard 100-µs click duration with our hardware). Clicks were timed according to a Poisson point process with a rate of 44.1 clicks/s. The timing of one click had no correlation with the timing of any other click in the train, rendering the sequence spectrally white in the statistical sense. A pair of 30-s sequences was created and presented dichotically 20 times to each subject, meaning that 26,460 clicks contributed to each ear’s response. The responses presented herein are the sum of the monaural responses. Clicks were presented at 75-dB peak-to-peak equivalent SPL (i.e., the amplitude of clicks matched the peak-to-peak amplitude of a 1-kHz sinusoid presented at 75-dB SPL).

While no previous study has used exactly this type of click timing, several have used various types of pseudorandom sequences (Burkard et al., 1990; Thornton and Slaven, 1993; Delgado and Ozdamar, 2004; Holt and Özdamar, 2014). Uniformly, these studies find that the ABRs from randomized versus periodic click trains are highly similar at the same stimulation rates. Random timing has two main benefits over the much more common periodic timing: (1) the analysis window for the response can be extended arbitrarily to any beginning and end point without fear of temporal wrapping; and (2) no high-pass filtering is necessary to remove the strong frequency component at the (periodic) presentation rate, because it does not exist. A third benefit, specific to this study, is that the same linear systems analysis could be done to compute the speech-derived and the click-evoked ABR, yielding a more direct comparison between the two. Figure 1*B*,*E*,*H* shows part of a Poisson click train in the same manner that Figure 1*A*,*D*,*G* do for speech.

To be sure that the click paradigm we used yielded results matching standard ABRs evoked with periodic click trains, we also collected ABRs using periodic click trains of the same rate of 44.1 clicks/s, presented diotically. Periodic trains were also presented in 20 epochs of 30 s, yielding the same total sweep count of 26,460. The periodic click train stimulus is shown in Figure 1*C*,*F*,*I*.

### Data analysis

Responses to both speech and click train stimuli were found through deconvolution, in a manner broadly similar to previous papers focused on cortical activity (Lalor et al., 2009; Lalor and Foxe, 2010). The essence of deconvolution is determining the impulse response of a linear time-invariant system given a known input (here, the processed continuous speech signal) and a known output (here, the recorded scalp potential). The methods in this study vary from previous ones in the recording parameters and preprocessing steps, but otherwise use essentially the same mathematical principles.

#### Speech stimuli preprocessing

Before we could derive the speech response, we needed to calculate the regressor from the audio data. The auditory brain is mostly agnostic to the sign of an acoustic input, as evidenced by the high degree of similarity between evoked responses to compression versus rarefaction clicks (Møller et al., 1995). For this reason, some sort of rectifying nonlinearity applied to the input speech is needed as a preprocessing step. We used half-wave rectification. Specifically, we performed all analyses twice, once keeping the positive peaks, and then a second time keeping the inverted negative peaks, and then averaged the resulting responses, in a process akin to the compound peristimulus time histogram used by Pfeiffer and Kim (1972). This alone significantly reduced, but did not eliminate, stimulus artifacts, similar to the common technique of alternating polarity in the click-evoked ABR (Hall, 2006). Further artifact reduction steps are described later in this section. Following rectification, the data were downsampled from 24,414 Hz to the EEG recording rate of 10,000 Hz.

#### Click train preprocessing

Owing to its extreme sparsity, downsampling a click train using standard methods would result in significant signal processing artifact, i.e., Gibbs ringing. We instead used the list of click times from the original click train (24,414-Hz sampling rate) and created a click train at 10,000-Hz sampling rate by placing unit-height single-sample impulses at the closest integer indices corresponding the original click times.

When the input to a system has a white power spectrum, the system’s impulse response can be determined as the cross-correlation of the input and output. For a click train, which is essentially a series of unit-height single-sample impulses, the deconvolved impulse response becomes equivalent to the click-triggered average, which is how ABRs are usually calculated. This results in a convenient parity between the typical averaging methods used for ABR and the deconvolution used here. In other words: rather than using a completely new mode of analysis for ABR (deconvolution), we have instead generalized the methods already in use to be appropriate for arbitrary stimuli, beyond click trains.

#### EEG preprocessing

EEG data were first high-pass filtered at 1 Hz (first-order Butterworth), and then notch filtered at 60, 180, and 300 Hz with 5 Hz wide second-order infinite impulse response notch filters, designed with the *iirnotch* function of the SciPy python package (RRID:SCR_008058). Because of the continuous nature of the stimuli, no epoch rejection was done. Instead, any time the EEG potential crossed ±100 µV, a 1-s segment of the response was zeroed, centered around the offending sample, removing it from the calculation. 100 µV is a larger rejection threshold than most EEG studies use, which was necessary because the EEG data had higher power due to the minimal filtering that was applied (high-pass at 1 Hz). Zeroing portions of an epoch slightly reduces its energy. So that the amplitude of the calculated response was not affected, the EEG data for each epoch was multiplied by a corrective gain factor *g*_r_:

$$g_{r}=N/(N–N_{r}),$$

where *N* is the total number of samples in the epoch and *N_r_* is the number of rejected samples. After filtering and resampling, the data were segmented into epochs that started with the stimulus onset and ended 100 ms after the stimulus (epochs were thus 64.1 s long for speech stimuli and 30.1 s long for clicks). With these parameters, a median of 1.1% (0.3–2.3% interquartile range) of data were rejected from each subject’s EEG recordings.

#### Stimulus artifact removal

EEG recordings from some subjects showed stimulus artifacts, resulting from electromagnetic “leakage” of the headphone driver to the EEG system. We developed a protocol for removing these artifacts which involved estimating the artifact and then subtracting it from the EEG recording. For each epoch, we first computed the discrete Fourier transform (DFT) of the stimulus (the raw stimulus audio, not the rectified stimuli) and the EEG recording. We then divided the EEG DFT by the stimulus DFT and computed the inverse DFT of the quotient. We then cropped that signal (which is effectively the estimated impulse response that describes the electromagnetic leakage as a system) to the lags in a 10-ms window centered around the artifact at –0.9 ms. Because this 10-ms impulse response was empirically estimated from the data, and was elicited by a stimulus with very little energy below ∼100 Hz, it contained a high level of low-frequency noise. This noise was removed by fitting a sixth-order polynomial to the estimated impulse response and subtracting that fit from the signal itself. The polynomial order was chosen as the lowest that removed artifacts clearly (by visual inspection) unrelated to the impulse response. Because the noise was so much larger than the true impulse response (and was largely made of frequencies absent from the speech signal), the result of this subtraction was a clear impulse response with little low-frequency noise remaining. This process was completed for every epoch individually, the average impulse response for each subject was computed across all epochs, and that average was multiplied by a 10-ms Hann window. Finally, for each epoch, the stimulus was convolved with the computed artifact impulse response, and the resulting signal was subtracted from the EEG recording.

The stimulus artifact was only removed from the speech-derived responses. These steps were not necessary for click-evoked responses because those artifacts manifest as single sharp spikes before 0 ms latency.

It should be noted that there exist simpler ways to eliminate or mitigate stimulus artifacts. The simplest is electromagnetic shielding around the headphone drivers. Alternating the polarity of the speech stimulus should also significantly reduce stimulus artifacts in future experiments. This could be done at the level of the 64-s epochs, or it could be done at the word or phrase level, as long as the phase inversions were hidden by silent gaps in the speech. However, these methods must be implemented at the time of the recording, and were not here, which is why the signal processing steps described above were used.

#### Response calculation

We used linear least-squares regression to calculate the responses, as in previous work (Lalor et al., 2009). The response was considered to be the weights over a range of time lags that best approximated the EEG output as the weighted sum of the input stimulus regressor over those lags. For the sake of computational and memory efficiency, the stimulus autocorrelation matrix and stimulus-response cross-correlation were both calculated via their Fourier counterparts using frequency-domain multiplication. These specific methods have been incorporated into the mne-python package (Gramfort et al., 2013; RRID:SCR_005972). The stimulus regressors were sufficiently broadband such that no regularization was necessary, so none was used (had there been near-zeros in their amplitude spectra this would not have been the case). The response weights were calculated over the range of lags spanning −150 to 350 ms. After the response was calculated, it was low-pass filtered at 2000 Hz (first-order Butterworth). For the speech stimuli, the response to each narrator was calculated separately, and then averaged to calculate each subject’s speech-derived response. The stimulus regressors were sufficiently broadband such that no regularization was necessary, so none was used (had there been near-zeros in their amplitude spectra, this would not have been the case).

#### Speech-derived response amplitude normalization

Auditory onsets elicit much larger responses than ongoing stimulus energy due to adaptation (Thornton and Slaven, 1993). However, this nonlinear adaptation is not accounted for by the linear regression. For that reason, the raw speech-derived responses, for which the majority of the stimulus energy can be considered “ongoing,” were much smaller than the click-evoked responses, whose stimuli are essentially a series of onsets. To correct for this, we computed a single empirical subject-specific normalization factor, *g_n_*, that put the speech-derived responses in a similar amplitude range as the click-evoked ones:

$$g_{n}=E_{i}\left(\sigma_{c}{}_{,i}\right)/E_{i}(\sigma_{s}{}_{,i}),$$

where *σ_c,i_* is the SD of subject *i*’s click-evoked response in the range of 0–20 ms, *σ_s_*_,_*_i_* is the same for the speech-derived response, and *E_i_* represents the mean over subjects. All speech-derived responses shown in microvolts have been multiplied by *g_n_*. In our study *g_n_* had a value of 28.2, but it must be stressed that this value depends on the unitless scale chosen for storing the digital audio (ours had a root-mean-square amplitude of 0.01), and is thus not suitable for use in other studies. For this reason, no direct amplitude comparisons were made between click- and speech-derived responses. Instead, we computed correlations (which do not depend on scaling factors) of their morphologies within subjects, as well as their Wave V latencies and amplitudes across subjects.

### Standard ABR measurement

The ABR to the periodic click trains was calculated through traditional averaging rather than regression. The raw data were notch filtered to remove line noise and low-pass filtered at 2000 Hz as described above. However, the high-pass filter was different: a causal second order Butterworth filter with a cutoff of 150 Hz was used to be consistent with standard practice and to generate a canonical waveform (Burkard et al., 2006; Hall, 2006). The response to each click presentation was then epoched from −3 to 19.7 ms, which was the longest window allowed by the periodic click rate of 44.1 clicks/s before temporal wrapping occurred. Filtered epochs were rejected if the peak-to-peak amplitude exceeded 100 μV.

## Results

### Poisson click trains yield canonical ABRs

**Figure 2.**
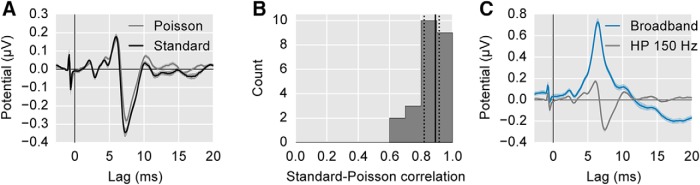
Comparison of ABR to standard periodic click trains and Poisson click trains. ***A***, The average ABR waveform evoked by the standard, periodic click train at 44.1 clicks/s (black) and the pseudorandom Poisson click train (gray; 44.1 clicks/s overall rate). Areas show ±1 SEM. Both responses are high-pass filtered at 150 Hz. The spike at −1 ms is a stimulus artifact, and occurs before 0 ms to compensate for the 1 ms tube delay of the earphones. ***B***, The histogram of per-subject correlation coefficients between the standard and Poisson click-evoked ABRs. Solid/dotted black lines show median/quartiles. ***C***, Comparison of the Poisson click-evoked ABR with 150-Hz high-pass filtering (gray) and without (i.e., broadband; blue). The latter is used as the benchmark response for the remainder of the study.

Responses to Poisson click trains were used as the benchmark to which the speech-derived responses were compared. Although similar types of pseudorandom stimuli have been used in the past, it was important to confirm that these specific stimuli used here provided canonical ABR waveforms. The grand average periodic and Poisson click trains are shown overlaid in Figure 2*A* (both shown high-pass filtered at 150 Hz). To quantify their similarity, we computed Pearson’s correlation coefficient between the two waveforms for each subject between lags of 0 and 19.7 ms. The median correlation was 0.89 (interquartile range 0.82–0.92), indicating a very high degree of similarity. The histogram of correlations is shown in Figure 2*B*.

Figure 2*C* shows the average Poisson click-evoked response under two filtering conditions: (1) high-pass filtered at 150 Hz (Fig. 2*A*); and (2) broadband (high-passed at 1 Hz as described in Materials and Methods, EEG preprocessing). The latter will be used henceforth as the click-evoked ABR to which the speech-derived ABR is compared. It is thus important to note that although these responses seem to have morphologic differences from the “standard” ABR, that is simply because using pseudorandom click timing obviates the need for high-pass filtering, and that filtering was bypassed in the interest of comparing the whole responses. The wideband responses we obtained here using Poisson click trains were highly similar in shape, amplitude, and latency to previous wideband (5-Hz high-pass) ABRs obtained using low rate (11 Hz) periodic clicks (Gu et al., 2012), and were much more efficient to obtain.

### Early speech-derived responses exhibit brainstem response characteristics

**Figure 3.**
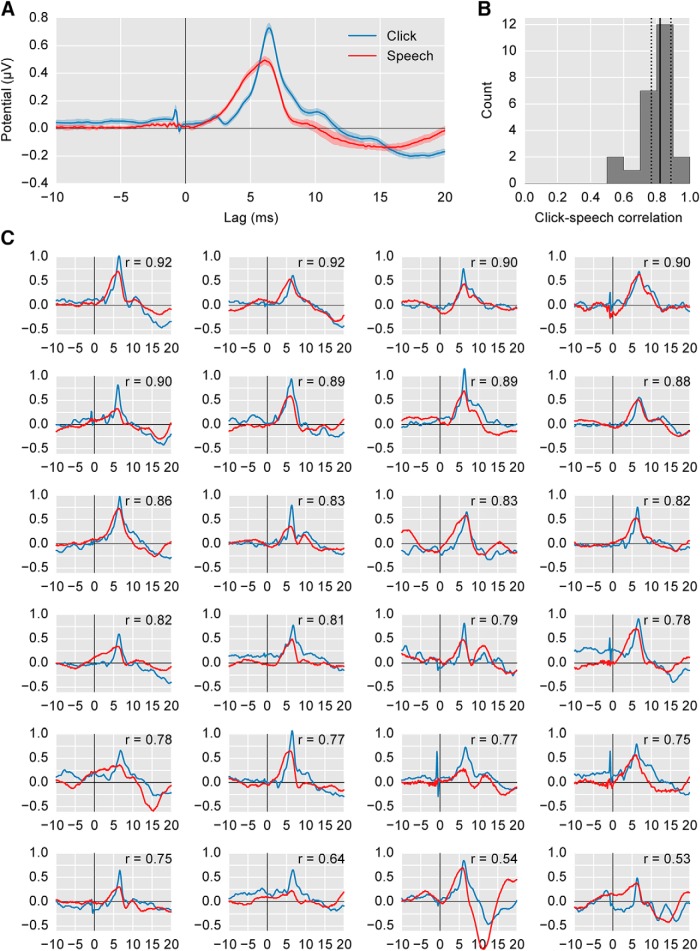
Comparison of click-evoked responses (blue) with speech-derived responses (red). ***A***, The average waveform across subjects (areas show ±1 SEM). ***B***, The histogram of correlation coefficients between the click-evoked and speech-derived stimuli for each subject. Solid/dotted black lines show median/quartiles. ***C***, Individual subject responses, sorted by descending correlation coefficient. The correlation is shown in the upper right corner.

Broadly speaking, there were strong similarities between the early (<20 ms) click-evoked and speech-derived responses (Fig. 3*A*). In this latency range, responses are likely to progress from brainstem to thalamus and primary auditory cortex as latency increases. We will first make whole-waveform comparisons, and then consider specific canonical ABR components.

To compare the overall waveforms, we computed Pearson’s correlation coefficient of the speech- and click-evoked waveforms for each subject in the range of 0–20 ms (Fig. 3*B*). The median correlation coefficient was 0.82 (interquartile range 0.77–0.89). Figure 3*C* shows each subject’s click- and speech-derived response, in descending correlation order. In our speech-derived responses, Waves I–IV were “smeared” together. However, we found a clear Wave V in individual subjects’ responses as well as the grand average. Wave VI was also visible in the grand average, but was less consistent at the individual-subject level.

**Figure 4.**
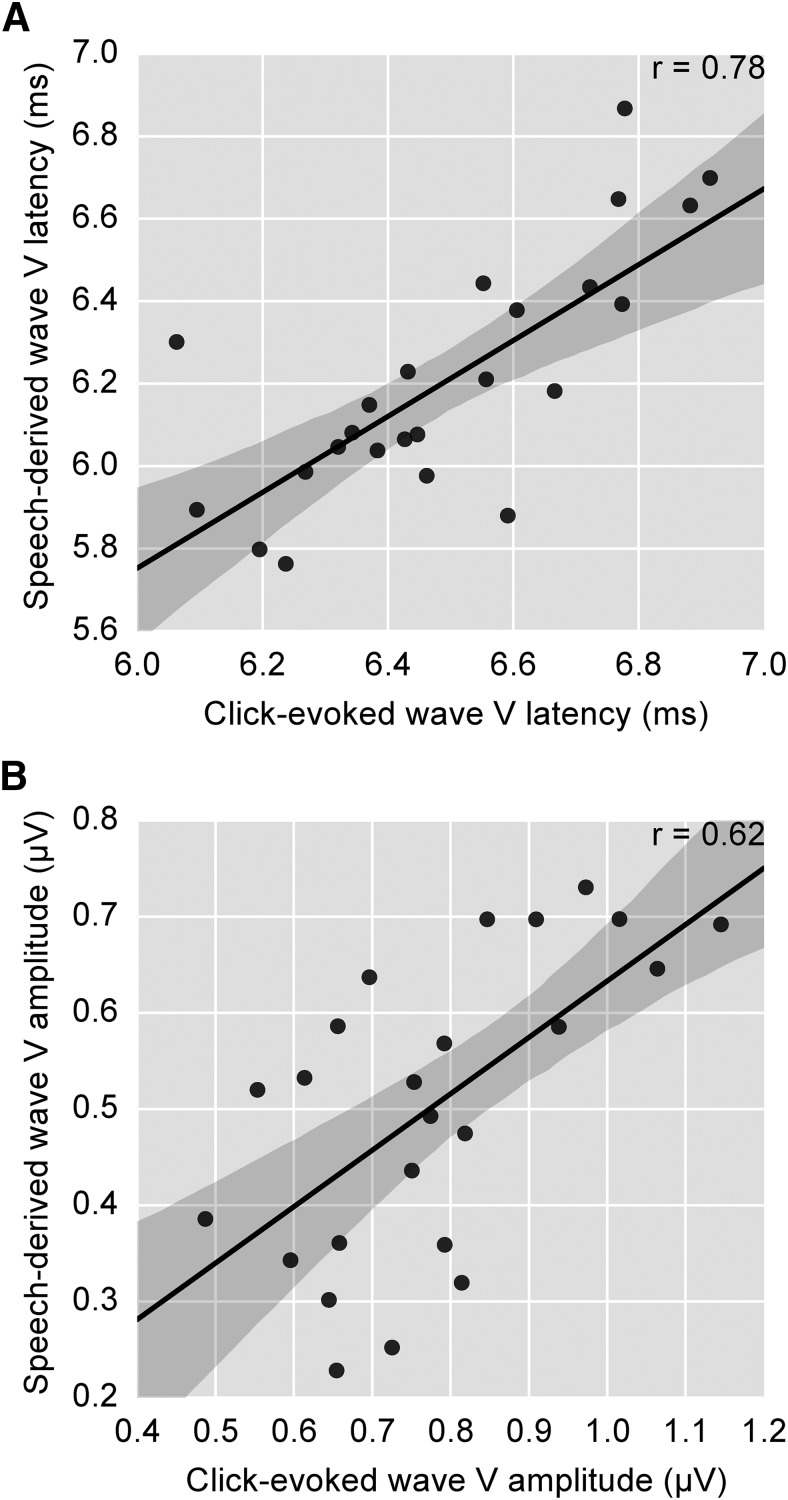
Correlation of speech-derived and click-evoked Wave V latencies (***A***) and amplitudes (***B***) across subjects. Because the click-evoked Wave V is known to be subcortical, the strong correlations across subjects point to brainstem neural generators for the speech-derived response as well. Points have been jittered slightly to prevent visual overlap. Regression lines are shown with the 95% confidence interval shaded.

We identified Wave V by low-pass filtering at 1000 Hz with a zero-phase filter and finding the peak of the waveform in the 5–7 ms range. For the click-evoked responses, Wave V was present for all subjects, with a latency of 6.50 ± 0.25 ms (mean ± SD). For speech-derived responses, Wave V was present for all subjects, with a latency of 6.17 ± 0.31 ms. As shown in Figure 4*A*, the click-evoked and speech-derived Wave V latencies were highly correlated across subjects (*r* = 0.78, *p* = 7 × 10^−6^, Pearson’s product-moment). The peak amplitudes of the speech-derived and click-evoked Wave V were also correlated (*r* = 0.62, *p* = 0.0011; Fig. 4*B*). These correlations strongly suggest that the click-evoked and speech-derived ABR have common neural generators.

### Speech responses across talkers are similar but not identical

**Figure 5.**
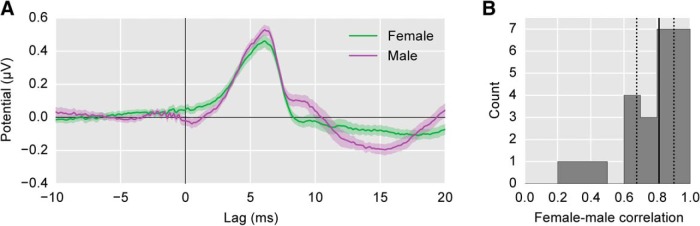
Comparison of female-narrated responses (green) with male-narrated responses (purple). ***A***, The average waveforms across subjects (areas show ±1 SEM). ***B***, The histogram of correlation coefficients between the female-evoked and male-evoked stimuli for each subject. Solid/dotted black lines show median/quartiles. The speech-derived ABRs from the male and female narrators show strong similarities, but are not identical, indicating some talker dependence.

One important question is whether the speech-derived response maintains its morphology independent of the specific input stimulus, or if it depends on the specific narrator. To investigate this, we split the responses to male- and female-narrated trials and compared them to determine the role that the difference in the narrators’ input spectra might play. The grand average waveforms for the two narrators are of the same magnitude and overall shape, despite the differing spectra of their input stimuli (Fig. 5*A*). The median female-male correlation coefficient was 0.81 (interquartile range 0.68–0.90; Fig. 5*B*).

While perfect overlap would be indicated by correlation coefficients of 1.0, splitting the data in half (namely, into male- and female-narrated epochs) adds noise to each of the responses. To put the male-female correlation coefficients in context, we can split the data a different way and compare. We split the data into halves consisting of the even versus odd trials, which contained the same number of male and female epochs (i.e., each split contained 10 male and 10 female trials, distributed evenly in time across the recording session). We then compared those waveforms as above. The median correlation coefficient between splits was 0.89 (interquartile range 0.78–0.92). We compared the male-female split coefficients to these arbitrarily split coefficients, and found a significant difference (*t*_(23)_ = 68, *p* = 0.019, Wilcoxon signed-rank test). This indicates that while the responses to female and male-uttered speech are indeed similar, there is still some dependence on the stimulus.

### Sufficient SNR was attained for all subjects

**Figure 6.**
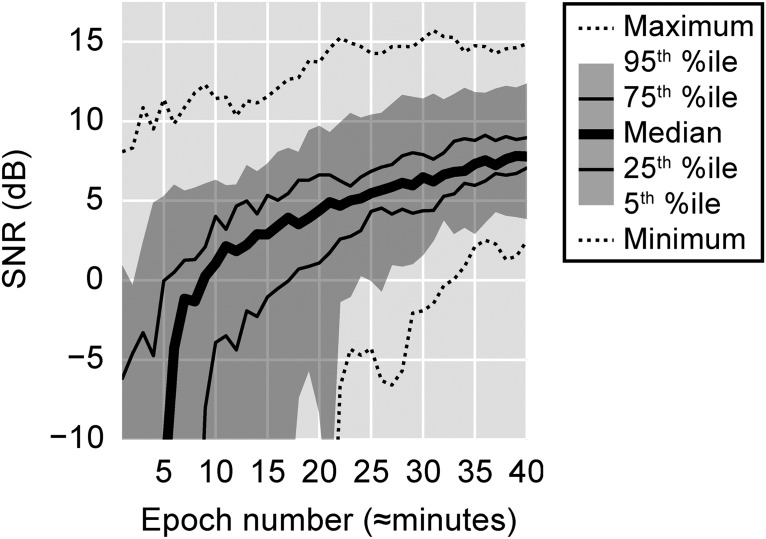
SNR as a function of data accumulation. SNR was calculated for each subject using the mean of all the data up to each recording epoch by computing the variance in the ABR time interval 0–20 ms, and in the prestimulus noise interval −125 to −10 ms.

A measure’s usefulness decreases with the amount of time required to obtain it. SNR generally increases with additional data, and is thus a function of recording time. To assess SNR, we first computed the cumulative average response up to each of the 40 recording epochs, for each subject. We then computed the SNR, in decibels, as

$$\text{SNR }=\text{ 1}0{\text{ log}}_{\text{1}0}[\left(\sigma^{\text{2}}{}_{\text{ABR}}-\sigma^{\text{2}}{}_{\text{noise}}\right)/\sigma^{\text{2}}{}_{\text{noise}}],$$

where *σ* ^2^_ABR_ and *σ* ^2^_noise_ are the variances of the response in the lag intervals from 0-20 ms and −125 to −10 ms, respectively. The results are plotted in Figure 6.

Experimental demands differ, but an SNR of 0 dB or better typically allows a response to be easily seen and inspected. For these 24 subjects, 50% achieved that threshold after 9 epochs, 75% after 17 epochs, 95% by 27 epochs, and all by the end of the thirty-third epoch. These data are shown in Figure 6. Taken as a whole, they confirm that the speech-derived ABR can be measured to a useful SNR in reasonable durations. Recording times should also be short enough that multiple conditions can be tested in a single experimental session. The median SNR’s evolution over time also generally aligned with the theoretical expectation of +3 dB per doubling of recording time: after 10, 20, and 40 epochs it was 1.1, 4.4, and 7.8 dB, respectively.

We also sought to address the stability of the speech-derived responses over time. To do so, we split the data into halves, epochs 1 through 20 and 21 through 40, and compared the responses (female- and male-narrated trials distributed evenly throughout each of these halves). Across subjects, the median correlation was 0.86 (interquartile range 0.81–0.92). These high correlations suggest responses were stable over the session. They are very similar to the even-odd trial split correlations, which would be less affected by a response that changes or drifts over the recording session, which had a median of 0.89 (0.78–0.92).

## Discussion

### Early speech responses are interpretable as ABRs

The major goal of this work was to study the response of the human auditory brainstem to naturally spoken, continuous speech. We computed the speech-derived responses using regression and validated them against click-evoked responses. Comparison of the speech-derived and click-evoked ABR revealed a high degree of morphologic similarity between waveforms, similar overall Wave V latencies, and a strong correlation between speech-derived and click-evoked Wave V latency and amplitude across subjects. Taken together, these results show that the speech-derived ABR developed here is just that: the response of the auditory brainstem to naturally uttered speech. Note, however, that the goal of this study was not to replace the click-evoked ABR, it was to allow the ABR to be measured in response to natural speech stimuli presented in the context of an engaging behavioral task.

Incoming acoustic information travels up the auditory pathway in an initial feedforward sweep, from brainstem to thalamus to cortex. Because the response calculated here is broadband, distinct components over the range of latencies were preserved. We can thus “localize through latency” and logically conclude that the peak in the response at ∼6 ms has subcortical origins, because it is too soon after the stimulus to be cortical, where the earliest estimated latencies are 11–14 ms (van Wassenhove and Schroeder, 2012). This eschews the problem of source mixing when attempting to determine brainstem activity through spatial means, such as beamforming and dipole fits. However, as discussed below, our method does not preclude those analyses, rather it complements them and facilitates their use, particularly at longer latencies where sources have cortical origins more appropriate for spatial filtering.

### Speech-derived ABR facilitates new studies of brainstem processing under natural conditions

The motivation for this work was to allow the study of auditory brainstem activity in human listeners without stimulus limitations. To demonstrate the technique’s utility, in this section we propose two important questions whose corresponding experiments are specifically facilitated by the new methods.

Natural speech has rich spectrotemporal structure. It also has semantic and connotative contents that transcend its acoustics, which cannot be ascertained by a listener unfamiliar with the language. But until now, studies of brainstem speech processing have been limited to simple repeated stimuli, and studies using natural speech have been mostly limited to the later potentials corresponding to the cortex. One exception to this is a recent study by Forte et al. (2017), whose central question is the first one discussed below.

#### Example experiment 1. Does selective attention to one of two competing speech streams affect the brainstem’s response to those streams?

A fundamental question in auditory neuroscience is how the brain selects one sound of interest from a mixture of several [first described by Cherry (1953) as the Cocktail Party Problem]. A number of studies have shown that in the cortex, the representation of a natural speech stream attended by subjects is greater than that of one to be ignored (Mesgarani and Chang, 2012; O’Sullivan et al., 2014). Many brainstem nuclei receive efferent projections from the cortex, or from higher nuclei of the brainstem, which suggests the possibility of cortical modulation of subcortical processing playing a role in selective attention (Terreros and Delano, 2015).

The present technique can be applied to studies using the same paradigms as previous cortically-focused ones: present two speech streams, ask the listener to attend to one, and calculate the speech-derived response from each stream. An effect of attention in the brainstem would manifest as a stronger response to the attended stream.

The strength of such a paradigm is that it is much more likely to engage attentional mechanisms due to its ecologicalal validity, whereas asking subjects to attend to one stream of clicks but not the other may not. The present technique allows specific measures of brainstem processing (e.g., Wave V amplitude and latency) to be reported as a function of attention. One recent study using natural speech has suggested a subcortical effect of attention (Forte et al., 2017). These results are promising, but because the responses are akin to a continuous FFR and the waveforms do not recapitulate the canonical ABR, the specific origins of the effect are harder to verify.

#### Example experiment 2. Does understanding speech affect its representation in the brainstem?

The brainstem, in particular the inferior colliculus, is very important to speech processing (Carney et al., 2015). It is not known if the processing in the brainstem is purely acoustical, or if it is affected by higher order (e.g., semantic) processing of the speech. With the speech-derived ABR it is possible to test that. A basic design to do so would involve two sets of subjects that spoke and understood only one of two separate languages (*A* and *B*). Speech stimuli from both languages would be presented to both groups, and the speech-derived ABR measured.

If, e.g., language *A* speakers showed a Wave V amplitude that was bigger for language *A* than language *B*, and language *B* speakers showed the opposite, then an effect of speech comprehension on brainstem processing would be indicated. An additional advantage of the technique is that if the speech-derived ABR did not show an effect, then the analysis window could be extended to show cortical processing as well (see next section), so that the first level at which comprehension does affect the response could still be investigated.

It is not possible to test effects of comprehension with click stimuli, because clicks cannot be understood. This example makes manifest the distinction between the click-evoked ABR and the speech-derived ABR developed here. While the present study has been aimed at showing the two responses’ neural origins are the same, their experimental applications are quite different.

### Subcortical and cortical responses are available simultaneously

While the focus of this work is on the brainstem and midbrain responses, these methods can be used to measure both subcortical and cortical activity. Simultaneous subcortical and cortical measurements are possible with the cABR (Skoe and Kraus, 2010), but the differing parameters for number of trials and inter-stimulus interval needed mean that recording paradigms can be very long. Work aimed at optimal parameters for simultaneous subcortical-cortical recordings has been successful (Bidelman, 2015), but still necessarily results in compromises. The present methods allow simultaneous measurement with no additional recording time and no limitations on the response window due to inter-stimulus interval.

**Figure 7.**
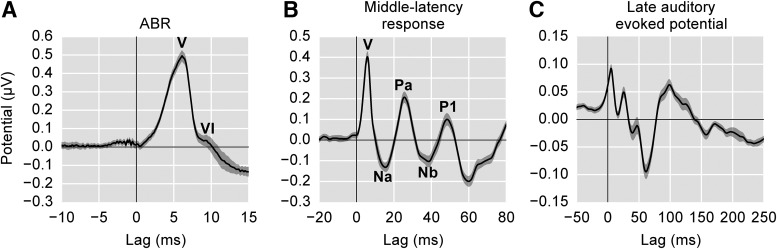
Changes to the range of lags and filtering parameters allow early, middle, and late responses to be analyzed from the same recording. ***A***, The average speech-derived ABR with Canonical Waves V and VI labeled. ***B***, The middle latency response with its canonical waves labeled (low-pass frequency: 200 Hz). ***C***, The late auditory evoked potential (low-pass frequency: 20 Hz). Due to adaptation, the amplitudes for the later waves are much smaller than typically seen in the event-related potential literature. These peaks are also not given canonical labels as in ***A***, ***B*** because their latencies do not directly correspond to the standard N1 and P2 peaks. Shaded areas show ±1 SEM.

This flexibility is illustrated in Figure 7, where the same average speech-derived response measured here is plotted three different ways. Figure 7*A* shows the speech-derived ABR, Figure 7*B* extends the window and employs a low-pass filter appropriate for viewing the middle latency response, and Figure 7*C* extends the time window further and lowers the low-pass frequency to accentuate late auditory evoked potentials of cortical origin. It is interesting to note in Figure 7*C* that, while cortical response amplitudes are generally thought of as being larger than the ABR, this is not the case when using continuous speech as a stimulus. This likely stems from the fact that there is significant cortical adaptation to a continuous stimulus, where typical event-related potential designs are careful to allow enough time between stimulus onsets to prevent adaptation. While the later peaks in Figure 7*C* are surely cortical in origin, their specific latencies do not perfectly match the canonical latencies of N1 and P2. It is not entirely clear why this would be the case.

While only one EEG channel was used here, there is no reason a full electrode montage could not be used, assuming one is available (along with considerable hard drive space). This would allow the simultaneous study of brainstem and cortical processing under natural conditions. Additionally, interactions between the two are also possible to study by adding interaction terms to the linear model. For example, a significant interaction between time-varying parietal α power and the size of the ABR could indicate a functional relationship between those areas.

### Filtering must be done carefully

It is common practice in EEG experiments to use zero-phase filters whose impulse responses are non-causal and symmetric about zero lag. This is done to preserve the latencies of the peaks and is appropriate in many cases. However, the strength of the present approach lies in using the latency of the response peaks to confirm their subcortical origin. If a non-causal filter is used to filter the EEG data, then it is possible that a peak at a latency corresponding to cortical activity (e.g., 25 ms) could affect the response waveform at brainstem latencies (e.g., 6 ms). This could have the result of erroneous findings that attribute cortical phenomena to subcortical nuclei. Thus, the following two guidelines are recommended for experiments specifically aimed at the auditory brainstem. First, EEG data should be filtered with causal filters. Second, when calculating regressors, any filtering that is done to the input stimulus should be anti-causal (i.e., with an impulse response that has non-zero values only at negative lags). The latter can be practically accomplished by reversing the signal in time, filtering it with a standard causal filter, and then reversing that result. Using causal filters will inevitably affect the latencies of peaks, but this can be mitigated by filtering sparingly (i.e., as broadband as the specific analyses will allow) with low-order filters, as was done here.

### Responses to arbitrary stimuli can be measured

For a spectrally rich but non-white stimulus like speech, an important step in deconvolution is whitening the input stimulus. For a linear system, two broadband stimuli with different spectra should yield the same impulse response. However, there is no such guarantee for a nonlinear system like the auditory system.

The present study suggests that it would be possible to use a range of stimuli to evoke responses with similar morphologies. First, we consider the main comparison: speech-derived to click-evoked ABR. Natural speech is different by almost any metric from Poisson click trains, and yet the responses that we find through regression are very similar (Fig. *3A*,*B*). Second, we consider the responses to female versus male speech. Males typically speak at a fundamental frequency about half that of females, due to relatively larger vocal folds. Such a difference, when estimating the response of a highly nonlinear system using linear methods, could have resulted in major differences in the response waveforms, but this was not the case (Fig. 5*A*,*B*). Taken together, it is reasonable to expect that the present technique could be applied to other real-world non-speech stimuli such as music or environmental sounds, as well any spectrally rich synthetic stimulus of interest in the lab.

Despite the similarity between responses to different stimuli, the differences (e.g., between the female and male speech-derived responses) do represent a caveat. In future studies, experimenters must be careful in making comparisons between responses across conditions that did not use identical stimuli. We suggest that these methods will be most useful in cases where the acoustic stimuli can be counterbalanced across conditions. While this is good practice in most studies, it is especially important here for drawing strong conclusions.

### Other regressors may offer improvements

An important difference between this study and those that came before it is choice of the regressor. Because the auditory system is fundamentally nonlinear (i.e., it responds with the same sign to both compression (positive) and rarefaction (negative) clicks), some sort of manipulation of the audio into an all-positive signal is needed. Previous studies have used the amplitude envelope (Aiken and Picton, 2008; Lalor and Foxe, 2010), spectrotemporal representations (Ding and Simon, 2009), and even dynamic higher-order features of speech (Di Liberto and Lalor, 2017).

Critically, the rectified speech audio used here is a broadband signal, which is what allows distinct ABR components at short latencies to be resolved in the derived response. There are many other transformations one could do, which will have important effects on the response waveform obtained. We piloted several (for example, “raising” the audio to be all-positive by adding it to its Hilbert amplitude envelope), but decided on the half-wave rectified audio due to its simplicity and the robustness of the responses it yielded. It is possible, likely even, that there are better transformations. One shortcoming of our approach is that no distinct Wave I was found, and all of Waves I–V were smeared together. An improvement in the regressor is the most likely route to addressing this, if it is indeed addressable, and will be a focus of future work.

### Conclusions and future directions

Here, we present and validate a method for determining the response of the auditory brainstem to continuous, naturally uttered, non-repeated speech. Speech processing involves a complex network that ranges from the earliest parts of the auditory pathway to auditory and association cortices. The technique described here facilitates new neuroscience experiments by making it possible to measure activity across the auditory neuraxis while human subjects perform natural and engaging tasks. These paradigms will allow study of the subcortical effects of language learning and understanding, attention, multisensory integration, and many other cognitive processes.

## Synthesis

Reviewing Editor: Satu Palva, University of Helsinki Decisions are customarily a result of the Reviewing Editor and the peer reviewers coming together and discussing their recommendations until a consensus is reached. When revisions are invited, a fact-based synthesis statement explaining their decision and outlining what is needed to prepare a revision will be listed below. The following reviewer(s) agreed to reveal their identity: Samira Anderson

All reviewers found your study very fascinating and sound. Also the questions raised by the previous reviews were all well addressed in the current submission and the decision to reorient this paper towards Methods was thought to improve its impact. There are few minor issues that should be corrected before publication that are found below.

Minor

--Kudos for using Shaun the Sheep, but the referencing might have gone astray somewhat:“(Shaun the Sheep (Season 1), n.d.)XXX”

-- In Figure 7, is the time axis for the late AEP really correct? The N1 peaking at about 65 ms, the P2 at about 95 ms? What am I missing? I see the statement on adaptation, but how can adaptation speed up latencies so drastically (rather than lengthen them)? I am not convinced by this argument. Also, the adaptation argument is only partly compelling: Other regression-based speech studies, also with such continuous stimuli (Lalor lab; Obleser lab; Simon lab), do find rather typical, ERP-like latencies for their response functions. So, I am confused all the more what we are actually looking at here, especially without topographies.

-- Both the abstract and the significance statement start with a statement on how speech processing“start in [nuclei of] the brain stem”. This strikes me as an odd and unnecessary claim. Why stop/start there? Why does it not start in the cochlea? At the eardrum? On the other hand, how speech-specific if anything that happens later on in cortex? By all accounts, nothing in the neural signal process is very much. I would probably simply skip that statement and save some words. The brain stem is cool enough and does not need that kind of touting.

## Author Response

Dear reviewers and Dr. Palva,

Thank you very much for your feedback on our revised manuscript. We are glad to see the positive feedback regarding our manuscript. Please see below for our responses to the final points.

All reviewers found your study very fascinating and sound. Also the questions raised by the previous reviews were all well addressed in the current submission and the decision to reorient this paper towards Methods was thought to improve its impact. There are few minor issues that should be corrected before publication that are found below.

We are happy to hear this-and we agree that embracing the Methods nature of the paper make it stronger.

--Kudos for using Shaun the Sheep, but the referencing might have gone astray somewhat:“(Shaun the Sheep (Season 1), n.d.)XXX”

Fixed.

-- In Figure 7, is the time axis for the late AEP really correct? The N1 peaking at about 65 ms, the P2 at about 95 ms? What am I missing? I see the statement on adaptation, but how can adaptation speed up latencies so drastically (rather than lengthen them)? I am not convinced by this argument. Also, the adaptation argument is only partly compelling: Other regression-based speech studies, also with such continuous stimuli (Lalor lab; Obleser lab; Simon lab), do find rather typical, ERP-like latencies for their response functions. So, I am confused all the more what we are actually looking at here, especially without topographies.

That is a fair question. The axis is correct, but we don't have a tidy explanation for why latencies of the later peaks using this method are different. We have noted this in the manuscript body and figure caption, and we have removed the peak labels from Fig. 7C, since there is no obvious correspondence to typical ERP peaks.

-- Both the abstract and the significance statement start with a statement on how speech processing“start in [nuclei of] the brain stem”. This strikes me as an odd and unnecessary claim. Why stop/start there? Why does it not start in the cochlea? At the eardrum? On the other hand, how speech-specific if anything that happens later on in cortex? By all accounts, nothing in the neural signal process is very much. I would probably simply skip that statement and save some words. The brain stem is cool enough and does not need that kind of touting.

Good point. We have changed these parts to say that the brainstem is important, while not making any claims of where speech processing starts or stops.

We have also made a change to the way we discuss an important recent study (Forte et al., 2017) and removed a reference to a previous study (Reichenbach et al., 2016) from the same group whose methods were improved upon by the new paper. These changes are in lines 62-66.

Thank you again for your efforts on our manuscript.

Sincerely,

Ross Maddox and Adrian KC Lee

## References

[B1] Abdala C, Folsom RC (1995) The development of frequency resolution in humans as revealed by the auditory brain‐stem response recorded with notched‐noise masking. J Acoust Soc Am 98:921–930. 7642831 10.1121/1.414350

[B2] Aiken SJ, Picton TW (2008) Human cortical responses to the speech envelope. Ear Hear 29:139–157. 18595182 10.1097/aud.0b013e31816453dc

[B3] Bidelman GM (2015) Towards an optimal paradigm for simultaneously recording cortical and brainstem auditory evoked potentials. J Neurosci Methods 241:94–100. 10.1016/j.jneumeth.2014.12.019 25561397

[B4] Burkard R, Shi Y, Hecox KE (1990) A comparison of maximum length and Legendre sequences for the derivation of brain‐stem auditory‐evoked responses at rapid rates of stimulation. J Acoust Soc Am 87:1656–1664. 2341669 10.1121/1.399413

[B5] Burkard RF, Don M, Eggermont JJ (2006) Auditory evoked potentials: basic principles and clinical application, Ed 1 Philadelphia: Lippincott Williams & Williams.

[B6] Carney LH, Li T, McDonough JM (2015) Speech coding in the brain: representation of vowel formants by midbrain neurons tuned to sound fluctuations. eNeuro 2. 10.1523/ENEURO.0004-15.2015 PMC4596011 26464993

[B7] Cherry EC (1953) Some experiments on the recognition of speech, with one and with two ears. J Acoust Soc Am 25:975–979. 10.1121/1.1907229

[B8] Coffey EBJ, Herholz SC, Chepesiuk AMP, Baillet S, Zatorre RJ (2016) Cortical contributions to the auditory frequency-following response revealed by MEG. Nat Commun 7:11070. 10.1038/ncomms11070 27009409 PMC4820836

[B9] Delgado RE, Ozdamar O (2004) Deconvolution of evoked responses obtained at high stimulus rates. J Acoust Soc Am 115:1242–1251. 15058345 10.1121/1.1639327

[B10] Di Liberto GM, Lalor EC (2017) Indexing cortical entrainment to natural speech at the phonemic level: methodological considerations for applied research. Hear Res 348:70–77. 10.1016/j.heares.2017.02.015 28246030

[B11] Ding N, Simon JZ (2009) Neural representations of complex temporal modulations in the human auditory cortex. J Neurophysiol 102:2731–2743. 10.1152/jn.00523.2009 19692508 PMC2777829

[B12] Ding N, Simon JZ (2012a) Neural coding of continuous speech in auditory cortex during monaural and dichotic listening. J Neurophysiol 107:78–89. 21975452 10.1152/jn.00297.2011 PMC3570829

[B13] Ding N, Simon JZ (2012b) Emergence of neural encoding of auditory objects while listening to competing speakers. Proc Natl Acad Sci USA 109:11854–11859. 10.1073/pnas.1205381109 22753470 PMC3406818

[B14] Forte AE, Etard O, Reichenbach T (2017) The human auditory brainstem response to running speech reveals a subcortical mechanism for selective attention. elife 6:e27203. 10.7554/eLife.27203 28992445 PMC5634786

[B15] Gramfort A, Luessi M, Larson E, Engemann DA, Strohmeier D, Brodbeck C, Goj R, Jas M, Brooks T, Parkkonen L, Hämäläinen M (2013) MEG and EEG data analysis with MNE-Python. Front Neurosci 7. 10.3389/fnins.2013.00267 PMC3872725 24431986

[B16] Grothe B, Pecka M (2014) The natural history of sound localization in mammals – a story of neuronal inhibition. Front Neural Circuits 8:116. 10.3389/fncir.2014.00116 25324726 PMC4181121

[B17] Gu JW, Herrmann BS, Levine RA, Melcher JR (2012) Brainstem auditory evoked potentials suggest a role for the ventral cochlear nucleus in tinnitus. J Assoc Res Otolaryngol 13:819–833. 10.1007/s10162-012-0344-1 22869301 PMC3505586

[B18] Hall JW III (2006) New handbook for auditory evoked responses, Ed 1 Boston: Pearson.

[B19] Holt FD, Özdamar Ö (2014) Simultaneous acquisition of high-rate early, middle, and late auditory evoked potentials. Conf Proc IEEE Eng Med Biol Soc 2014:1481–1484. 25570249 10.1109/EMBC.2014.6943881

[B20] Lalor EC, Foxe JJ (2010) Neural responses to uninterrupted natural speech can be extracted with precise temporal resolution. Eur J Neurosci 31:189–193. 10.1111/j.1460-9568.2009.07055.x 20092565

[B21] Lalor EC, Power AJ, Reilly RB, Foxe JJ (2009) Resolving precise temporal processing properties of the auditory system using continuous stimuli. J Neurophysiol 102:349–359. 10.1152/jn.90896.2008 19439675

[B22] L’Engle M (2012) A wrinkle in time. New York: Listening Library.

[B23] Mesgarani N, Chang EF (2012) Selective cortical representation of attended speaker in multi-talker speech perception. Nature 485:233–236. 10.1038/nature11020 22522927 PMC3870007

[B24] Møller AR, Jho HD, Yokota M, Jannetta PJ (1995) Contribution from crossed and uncrossed brainstem structures to the brainstem auditory evoked potentials: a study in humans. Laryngoscope 105:596–605. 10.1288/00005537-199506000-00007 7769942

[B25] O’Sullivan JA, Power AJ, Mesgarani N, Rajaram S, Foxe JJ, Shinn-Cunningham BG, Slaney M, Shamma SA, Lalor EC (2014) Attentional selection in a cocktail party environment can be decoded from single-trial EEG. Cereb Cortex 25:1697–1706. 24429136 10.1093/cercor/bht355 PMC4481604

[B26] Pfeiffer RR, Kim DO (1972) Response patterns of single cochlear nerve fibers to click stimuli: descriptions for cat. J Acoust Soc Am 52:1669–1677. 10.1121/1.1913301 4641371

[B27] Scott M (2007) The Alchemyst: the secrets of the immortal Nicholas Flamel, Book 1. New York: Listening Library.

[B28] Skoe E, Kraus N (2010) Auditory brainstem response to complex sounds: a tutorial. Ear Hear 31:302–324. 10.1097/AUD.0b013e3181cdb272 20084007 PMC2868335

[B29] Smith JC, Marsh JT, Brown WS (1975) Far-field recorded frequency-following responses: evidence for the locus of brainstem sources. Electroencephalogr Clin Neurophysiol 39:465–472. 52439 10.1016/0013-4694(75)90047-4

[B30] Starzak R, Sadler C (2007) Shaun the sheep (season 1). Bristol: Aardman Animations.

[B31] Terreros G, Delano PH (2015) Corticofugal modulation of peripheral auditory responses. Front Syst Neurosci 9 10.3389/fnsys.2015.00134 PMC4588004 26483647

[B32] Thornton ARD, Slaven A (1993) Auditory brainstem responses recorded at fast stimulation rates using maximum length sequences. Br J Audiol 27:205–210. 10.3109/03005369309076694 8241969

[B33] van Wassenhove V, Schroeder CE (2012) Multisensory role of human auditory cortex In: The human auditory cortex. Springer handbook of auditory research, pp 295–331. New York: Springer.

